# Femoral nerve palsy following kidney transplantation: A case report and review of the literature

**DOI:** 10.1002/iju5.12207

**Published:** 2020-07-29

**Authors:** Shuhei Yamada, Kiyohiko Hotta, Masahiko Takahata, Daiki Iwami, Yuki Sugito, Tatsu Tanabe, Naoya Iwahara, Nobuo Shinohara

**Affiliations:** ^1^ Department of Urology Hokkaido University Hospital Sapporo Japan; ^2^ Department of Orthopaedic Surgery Hokkaido University Hospital Sapporo Hokkaido Japan

**Keywords:** complication, femoral nerve palsy, kidney, self‐retaining retractor, transplantation

## Abstract

**Introduction:**

Femoral nerve palsy is a rare but serious complication of kidney transplantation. We report a case of femoral nerve palsy following kidney transplantation and conduct a review of the literature on this complication.

**Case presentation:**

A 35‐year‐old woman with end‐stage kidney disease, underwent kidney transplantation in the right iliac fossa. The day after the transplantation, she could not straighten her right leg. Physical examination revealed a paresis of her right quadriceps muscle. The patient’s sensation of her right thigh was also impaired. We diagnosed her with femoral nerve palsy caused by inappropriate compression from a self‐retaining retractor. Rehabilitation was started immediately. The patient’s motor weakness gradually improved, and the patient became able to walk independently 4 weeks later. However, the patient’s neuropathic pain sustained 6 months after her kidney transplantation.

**Conclusion:**

The improper use of self‐retaining retractors can lead to femoral nerve palsy in patients undergoing kidney transplantation.

**Abbreviations & Acronyms**
FN: femoral nerve
FNP: femoral nerve palsy
KT: kidney transplantation

**Keynote message**

We described a case of FNP after KT and reviewed the previous reports of FNP in kidney recipients. Improper use of self‐retaining retractors can lead to FNP in KT. Although the prognosis of motor function is favorable, neuropathic pain may persist. Therefore, careful attention to the direction and the depth of self‐retaining retractors is necessary during KT, and the use of retractors with a shallower blade is advised.

## Introduction

FNP after KT is a rare but serious complication that can compromise postoperative recovery and prolong the hospital stay.^1^, ^2^ FNP can cause gait disturbances due to motor dysfunction of the quadriceps muscle, resulting in deterioration of a patient’s quality of life.^3^

In this report, we describe the clinical course of a case of FNP after KT. Furthermore, we review previous reports of FNP in kidney recipients to determine the pathogenesis, prognosis, and proper surgical techniques for preventing FNP.

## Case presentation

A 35‐year‐old woman with end‐stage kidney disease due to focal segmental glomerulosclerosis underwent an ABO compatible preemptive KT in the right iliac fossa. Her body mass index was 18.7 kg/m^2^. She had low titer donor specific antibodies. The induction immunosuppression regimen included tacrolimus, mycophenolate mofetil, methylprednisolone, basiliximab, and rituximab. The total operating time was 5 h and 7 min, including a 1 h 31 min wait for the donor kidney. The surgical techniques are described in next part. The graft function after surgery was excellent. However, on postoperative day 1, the patient was not able to straighten her right leg. Physical examination revealed a paresis of her right quadriceps muscle. The patient’s sensation of her right thigh was also impaired. Computed tomography and magnetic resonance imaging showed no significant findings that could lead to FNP including hematoma or spinal disk herniation. We diagnosed her with FNP caused by inappropriate compression from a self‐retaining retractor during surgery. Rehabilitation was started immediately. The patient’s motor weakness gradually improved, and the patient became able to walk independently 4 weeks later. The patient began to complain of neuropathic pain as her sensory disturbance recovered. Despite the administration of pregabalin (200 mg/day), her neuropathic pain sustained 6 months after her KT.

### Surgical techniques

**Fig. 1:**
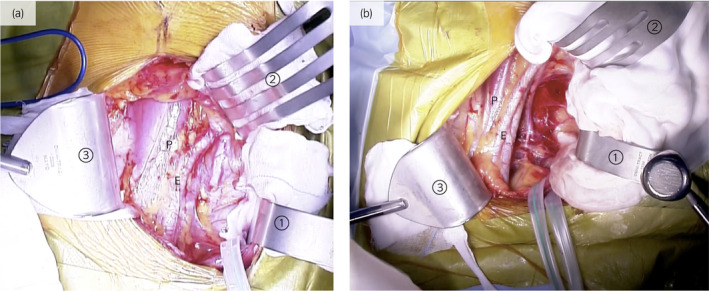
(a) Usual case: self‐retaining retractor deployment. We used three blades, in the mid‐medial (①), upper‐medial (②), and mid‐lateral directions (③). A folded surgical gauze is inserted between blades and the body to prevent tissue injury. P, psoas muscle; E, external iliac artery. (b) Current case. The mid‐lateral blade (③) to the retractor was directed more caudally than usual.

A lower quadrant curvilinear incision was made. A self‐retaining retractor with three blades in the mid‐medial, upper‐medial, and mid‐lateral directions was used. In this case, the mid‐lateral blade to the retractor was directed more caudally than usual (Fig. 1a,b). Arterial dissection was performed from the proximal common iliac artery to the distal external iliac artery. The internal iliac vein was ligated to mobilize the external iliac vein. The renal artery was anastomosed to the common iliac artery, and the renal vein to the external iliac vein.

## Discussion

**Fig. 2:**
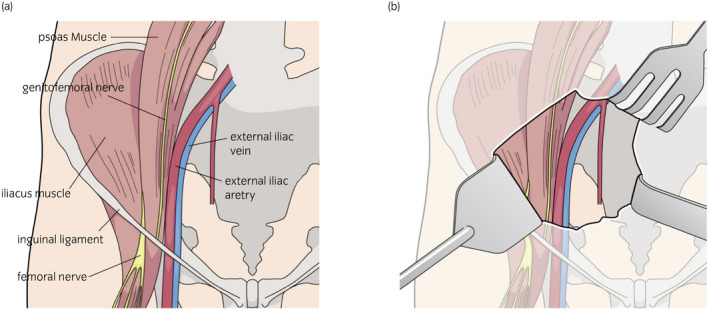
(a) Schema of the course of femoral nerve: femoral nerve comes out from the lateral border of psoas muscle 4 cm above the inguinal ligament. The femoral nerve descends in a shallow groove between the iliac and psoas major muscle. (b) Schema of the operative field in the current case: in the lower part of the psoas muscle, the femoral nerve is closer to the surface and can be compressed easily.

The FN is the largest branch of the lumbar plexus (L2 to L4), and is derived from the posterior divisions of the anterior primary rami of the second, third, and fourth lumbar spinal nerves. Formed within the psoas muscle, it comes out from the lateral border of this muscle 4 cm above the inguinal ligament, lying beneath the fascia on the iliacus.^2^ The FN descends in a shallow groove between the iliac and psoas major muscle (Fig. 2a). Therefore, the FN is vulnerable to damage by retractor injury due to its anatomical position. The lateral blades of the self‐retained retractor can easily compress the FN when use incorrectly. Furthermore, due to the low blood supply to the nerve in this area, any compression can easily lead to ischemic damage to the nerve.^4^ Previous studies have reported three mechanisms to explain the cause of FNP following KT: physical compression, traction or impinging damage, and ischemic injury of the FN.^1^, ^2^, ^5^

There are few published reports of FNP after KT. Our case and other reports of FNP after KT are summarized in Table 1. In retrospective studies, the reported incidence of FNP after KT varies from 0.14% to 8.4%.^1^, ^3^, ^6^, ^7^, ^8^, ^9^, ^10^ Likewise, in a prospective study,^11^ FNP after KT occurred in 4 of 184 recipients (2.2%). According to a prospective study by Nikoobakht *et al.*, the risk of FNP is higher in female patients and in those with a history of diabetes, although factors such as age, dialysis time, body type, anastomotic time, retractor time, and operation time had no effect on the appearance of FNP.^12^ A prospective study of FNP after hysterectomies suggested that the incidence of FNP is associated with the use of self‐retaining retractors.^13^

**Table 1:** Reported cases of post‐transplant femoral neuropathy

Case no.	Author	Gender	Age	Side	Lesion	Recovery
1	Vaziri *et al*.^6^	Female	30	Right	M, S	8 weeks
2		Female	22	Right	M, S	7 months
3		Male	51	Left	M, S	1.5 weeks
4	Yazbeck *et al*.^7^	Male	18	Right	M	1 month
5		Male	16	Right	M, S	M 1 week; S 2 months
6	Vogels *et al*.^8^	Male	5	Left	M, S	5 months
7		Male	16	Left	M, S	1 month, largely recovered
8		Female	12	Right	M, S	8 months
9	Sisto *et al*.^9^	Male	48	N/A	M, S	12 months
10		Male	29	N/A	M, S	M 2 months, S 6 months
11		Female	42	N/A	M, S	M 5 months, S 12 months
12		Male	63	N/A	M, S	44 months partial
13		Male	57	N/A	M, S	30 months partial
14		Male	40	N/A	M	3 months
15		Male	26	N/A	M, S	3 months
16	Sharma *et al*.^11^	Female	58	Right	M, S	3 months
17		Female	63	Right	M, S	6 months
18		Female	32	Right	M	9 months
19		Male	41	Right	M, S	6 months
20	Jog *et al*.^10^	Male	40	Right	M, S	4 months
21		Male	39	Left	M, S	6 months
22		Female	32	Left	M, S	6 months
23		Male	40	Left	M, S	6 months, impaired motor function
24		Female	18	Left	M, S	6 months
25	Van *et al*.^3^	Male	48	Left	M, S	3 months
26		Male	69	Right	M, S	1 year
27		Male	66	Left	M	Died 2 months after transplantation
28		Male	59	Right	M, S	1 year, hypoesthesia
29		Male	59	Right	M	3 months, partial
30	Kim *et al*.^1^	Female	42	Right	M	2 months
31		Female	61	Right	M	10 months
32		Female	49	Right	M	3 months
33		Male	54	Right	M	12 days
34		Female	26	Right	M	3 days
35	Current study	Female	35	Right	M, S	M 1 month, S 6 months
Overall		Male 21 Female 14	Median 40 (5–69)	Right 19 Left 9	M, S 25 M 10	M median 4 months (3 days to 44 months) S median 6 months (1–44 months)

M, motor function; N/A, not available; S, sensory function.

Motor function has good prognosis in most cases of FNP. Table 1 shows that recovery of motor function ranged from 3 days to 44 months (median 4 months). The patient in this study had good motor recovery. However, impaired sensation can persist and result in postsurgical pain. Recovery of sensory function ranged from 1 month to 44 months (median 6 months). Our patient continues to suffer from neuropathic pain 6 months after her KT. One systematic review reports that postoperative neuropathic pain can persist long term, which is distressing for patients and reduces their quality of life.^14^

In this case, the mid‐lateral blade to the retractor was directed more caudally than usual. In the lower part of the psoas muscle, the femoral nerve is closer to the surface and can be compressed easily (Figs 1b and 2b). Furthermore, the self‐retaining retractors are typically released while waiting for the donor kidney, but we forgot to do so in this case, leading to unnecessary compression on the femoral nerve for 90 min. Despite the results of a prospective study that showed no effect on appearance of FNP, it may cause ischemia and risk of FNP. In order to avoid FNP, a shallow blade should be used to provide traction of the lower lateral surgical field during KT.

## Conclusion

Improper use of self‐retaining retractors can lead to FNP in KT. Although the prognosis of motor function is favorable, neuropathic pain may persist. Therefore, careful attention to the direction and the depth of self‐retaining retractors is necessary during KT, and the use of retractors with a shallower blade is advised.

## Conflict of interest

The authors declare no conflict of interest.
